# EXERCISE MODALITIES AND OUTCOME MEASURES USED IN OLDER ADULTS AFTER HIP FRACTURE WITH OR WITHOUT SIGNS OF COGNITIVE IMPAIRMENT: A NATIONAL CROSS-SECTIONAL E-SURVEY OF 90 OUT OF 98 MUNICIPALITIES IN DENMARK

**DOI:** 10.2340/jrm.v58.44207

**Published:** 2026-02-11

**Authors:** Jan A. OVERGAARD, Morten T. KRISTENSEN, Lauren A. BEAUPRE, Kristian S. FREDERIKSEN, Søren T. SKOU

**Affiliations:** 1 Department of Rehabilitation, Lolland Municipality, Maribo; 2 Research and Implementation Unit PROgrez, Department of Physiotherapy and Occupational Therapy, Central and West Zealand Hospital, Region Zealand, Slagelse; 3 Center for Muscle and Joint Health, Department of Sports Science and Clinical Biomechanics, University of Southern Denmark (SDU), Odense; 4 Department of Physical and Occupational Therapy, Copenhagen University Hospital, Bispebjerg-Frederiksberg, Copenhagen; 5 Department of Clinical Medicine, Faculty of Health and Medical Science, University of Copenhagen, Copenhagen, Denmark; 6 Faculty of Rehabilitation Medicine–Physical Therapy, University of Alberta, Edmonton, Canada; 7 Danish Dementia Research Centre, Department of Neurology, Copenhagen University Hospital – Rigshospitalet, Copenhagen, Denmark

**Keywords:** hip fracture, cognitive impairment, exercise, outcome measures

## Abstract

**Objective:**

To investigate rehabilitation settings, exercise modalities, and assessments provided post-discharge to older adults with hip fracture with and without signs of cognitive impairment.

**Design:**

National cross-sectional e-survey.

**Subjects:**

Rehabilitation managers and development physiotherapists from all 98 Danish municipalities.

**Methods:**

Information was collected on rehabilitation after discharge across four settings: 24-h-care, home-based care, outpatient-healthcare-centers, and nursing-home-facilities.

**Results:**

Ninety municipalities (92%) responded. About half used standardized screening tools to guide rehabilitation, but only 4% screened for cognitive impairment. Rehabilitation was typically delivered by physiotherapists 1–2 times/week, lasting 5–12 weeks, with 24-h-care and nursing-home facilities settings offering shorter but more frequent sessions. Common exercise modalities included strengthening, balance, and functional tasks, where these were more used in hip fracture than hip fracture with signs of cognitive impairment. Patient-Reported-Outcome-Measures (PROMs) were infrequently used; the Patient-Specific-Functional-Scale and Numerical-Rating-Scale were most often used. Cognitive PROMs were rarely applied, except the Montreal-Cognitive-Assessment at 24-h-care. Performance-based tests were more widely used, particularly the 30s-sit-to-stand and Timed Up&Go tests.

**Conclusion:**

The survey had a high response rate. Few municipalities used cognitive screening tests and pain scales whereas performance-based testing was more predominant. The preferred exercise modality was functional exercise, used more often for patients with hip fracture than those with hip fracture and signs of cognitive impairment.

## LAY ABSTRACT

After hip fracture, patients need rehabilitation to regain their physical function. In Denmark, this mostly takes place in municipalities after hospital discharge, with the goal of returning to previous activity levels. However, little is known about the type and length of training, or which tests are used, especially for patients with memory difficulties. We asked all 98 municipalities in Denmark about their rehabilitation services for older adults with hip fracture, including those with signs of memory difficulties. Ninety municipalities responded. Most municipalities offered exercise programmes 1–2 times per week for 5–12 weeks, but programmes in nursing homes or 24-h settings were often shorter or more intensive. Physiotherapists mainly delivered the rehabilitation, and programmes often focused on strengthening, balance, and daily function. Tools to assess memory loss and patient-reported outcomes were rarely used, whereas physical performance tests like the sit-to-stand and walking tests were widely applied across all settings.

The worldwide number of hip fracture (HF) is rising with a predicted 1.9-fold increase in the incidence of HF rates from 2018 to 2050 (1), and with an increase in the incidence rates (per 1,000 citizens) in Denmark from 5.79 in 2019 to 6.18 in 2023 (2). Experiencing a HF can be disastrous for many older adults, leading to increased dependency and loss of function (3, 4). In addition, high morbidity and mortality is seen for patients with a fragility fracture of the hip (3).

Older adults with HF and signs of cognitive impairment (HF-SCI) including dementia represent up to 40% of all HF (5, 6). Even though this subgroup is relatively large, they are often not included in research (6). From the available evidence it is clear that patients with HF-SCI experience a substantial negative impact on outcomes (e.g., activities of daily living [ADL] and physical function [7–9]) and have a 2.7 times higher risk of dying than those without cognitive impairment (10).

Hall and colleagues (5) found in a qualitative study that very few patients with HF-SCI in the United Kingdom were labelled as not having potential for rehabilitation. However, patients with HF-SCI receive less rehabilitation than those without cognitive impairment (11, 12), which can have a massive negative personal and societal impact. Furthermore, physiotherapists face the clinical challenge that patients with HF-SCI may have difficulty following instructions and participating in rehabilitation (13). To further understand the reasons behind this apparent difference in rehabilitation, and to improve clinical practice and ensure equity along with individualized care, a detailed understanding of the rehabilitation provided is needed.

Treatment and rehabilitation are provided to all residing in Denmark through a publicly tax-funded healthcare system and delivery of services is shared between hospitals and municipalities. Specifically, hospitals provide surgery, medical management, and rehabilitation immediately post-surgery, and municipalities provide rehabilitation and ongoing supportive care after discharge from hospital. This collaborative model addresses both immediate medical needs and long-term well-being within the universal healthcare system (14). Over the last 7 years, the length of stay in hospitals among older adults with HF in Denmark has declined from a mean of 9 days in 2017 to 7 days in 2023 (2). This results in more complex health interventions in the municipalities than before, as almost all aspects of the rehabilitation after HF take place in the Danish municipalities after the patient is discharged from hospital. A study published a decade ago (15) provided some evidence on what was delivered in about half of the municipalities in Denmark to patients with HF but not on those with HF-SCI. Thus, what is currently being provided to patients with HF and HF-SCI in terms of specific exercise modalities and assessments in the specific settings within the municipalities across Denmark is unknown.

This study aims to investigate the rehabilitation settings, exercise modalities, and assessments provided to patients with HF and for those with HF-SCI in Danish municipalities.

## MATERIALS AND METHODS

### Questionnaire development and pilot-testing

The development of this questionnaire was based on a former cross-sectional national survey conducted in 2014 by Kronborg et al. (15) and reported according to Strengthening the Reporting of Observational Studies in Epidemiology (STROBE) statement for cross-sectional studies and the Checklist for Reporting Results of Internet E-Surveys (CHERRIES) (16, 17). We extended the questionnaire by including questions on patients with HF-SCI. In Danish municipalities, signs of cognitive impairment can be identified in several ways. Most often, the referral of patients with HF and a documented diagnosis of dementia to municipal rehabilitation from hospitals will include this information. In patients without a diagnosis of dementia, the municipality-based physio- or occupational therapist observes memory impairment or behaviours associated with cognitive impairment. To facilitate respondents’ evaluation of the intervention and testing of their older adults after HF with or without HF-SCI, we included a cover letter with the survey outlining our definition of cognitive impairment/dementia: “Cognitive impairment may manifest as difficulty understanding or failing to follow exercise instructions, inability to remember events (e.g., what happened yesterday, reflecting short-term memory deficits), or problems with communication (despite previously being able to speak and understand Danish)”.

The municipality settings, where rehabilitation is usually provided in Denmark, were divided into 4 categories: 24-h care facilities (24-H) where staff are available around the clock to provide care and rehabilitation (often a temporary residence before further “discharge” to final residence), home-based (HB) care where physical rehabilitation occurs in the patient’s own home, outpatient healthcare centres (OPC) where patients are transported from their homes for rehabilitation, and nursing home facilities (NHF) that serve as permanent residences for the most frail individuals. Further, we wanted to gain knowledge concerning the different kind of elements of exercise modalities provided, the provider, and duration of the intervention (frequency and minutes/week; number of weeks).

Kronborg et al. (15) provided information on the use of outcome measures in the municipalities, which we expanded to include a total of 25 commonly used patient-reported (PROMs) and performance-based outcome measures (Table SI). We asked about the use of these measures across all 4 settings (24-H, HB, OPC, and NHF) in patients with HF and with HF-SCI.

The questionnaire underwent 3 rounds of pilot testing with stakeholders to assess the relevance of its content, clarity, and comprehensibility of the questions. In the first pilot round, the questionnaire was sent to a manager and 3 development physiotherapists from 4 different municipal practices, mainly in the eastern part of Denmark. After incorporating their feedback, the revised questionnaire was distributed to a researcher (within the area of HF), 1 manager and a physiotherapist from another municipality in the western part of Denmark. Following further revision, the questionnaire was then programmed in a Research Electronic Data Capture (REDCap, Vanderbilt University, USA) web-based software platform (18, 19) and tested for user-friendliness by a Lolland Municipality based occupational therapist and a physiotherapist. All reviewers had extensive knowledge of the target group and the structure of the healthcare system, including the municipal system in Denmark. An English version of the questionnaire is provided as Appendix S1.

### Responder recruitment process

The first author (JAO) obtained contact information for municipal managers and development physiotherapists in municipality-based rehabilitation departments through online sources and a professional network. As post-hospital rehabilitation primarily occurs in municipalities, all 98 of Denmark’s municipalities were contacted by phone to ensure representation. Those who agreed received an e-mail with study details, a questionnaire guide, and a unique REDCap survey link. The guide specified that, if needed, respondents could consult relevant physiotherapists or occupational therapists within their own municipality, but that they had to provide a consolidated response to make it as representative as possible. Initially, all 98 municipalities agreed to participate.

### Data collection and management

The electronic survey was administered through REDCap at Region Zealand between 21 March and 31 May, 2023. Each municipality received a unique survey link on 21 March, followed by 2 reminder emails and follow-up phone calls to encourage participation.

Five municipalities requested to complete the survey on paper, later manually entered into REDCap. One municipality lacked data on 24-H rehabilitation and another on NHF. After data collection, all surveys were reviewed for completeness, with no imputation for missing responses.

Information on municipality-based rehabilitation after hospital discharge was collected for both cognitively intact patients with HF and those with HF-SCI. Data were gathered by examining the settings of delivery within the municipality (24-H, HB, OPC, NHF). Information on whether there was a formal description of the rehabilitation programme, the use of tools for stratification of rehabilitation or cognitive screening, the primary responsible profession for the rehabilitation, and the availability of extended rehabilitation options were also extracted for descriptive purposes.

### Data analysis

Survey responses were organized according to the weekly number of sessions, the duration of each session in minutes, and the overall length of the rehabilitation in weeks.

In addition, the following modalities of exercise were categorized into what was delivered in the 4 different settings:

- Warm-up (e.g., on a stationary bike, walking with rollator);
- Cardiovascular exercise (BORG [20] intensity level 14–15);
- Fitness (below BORG 14 intensity);
- Strength exercises;
- Strengthening exercises (defined as more than +15 repetitions per exercise);
- Balance exercise (static or dynamic);
- Skill/functional tasks (flights, in/out of bed, up from floor);
- Exercises of relevant activities of daily living (ADL) (bathing, getting dressed, eating/drinking);
- Outdoor walking (with or without walking aid);
- Other.

The PROMs and performance-based scores/outcome measures used in the 4 different settings and groups were also extracted from the questionnaires.

The data were analysed with the IBM Statistical Package for the Social Sciences (SPSS) program version 29 (IBM Corp, Armonk, NY, USA) and Microsoft 365 Excel program (Microsoft Corp, Redmond, WA, USA) using descriptive statistics and presented as absolute data (numbers and/or percentages). A χ^2^ or Fisher’s exact test was used as appropriate to illustrate differences with a level of significance of *p* < 0.05.

### Ethics

The study was approved by the Regional Scientific Ethical Committee in Region Zealand (approval number SJ-997) and by the Danish Data Protection Agency (Forskningsfortegnelsen, Region Zealand, approval number REG-096-2022, and the University of Southern Denmark Legal Office – SDU RIO, number 11.817). In the distributed materials, respondents were explicitly notified that by completing the questionnaire they consented to their data being used in the study, that their response would be managed anonymously and that only aggregated results would be presented.

## RESULTS

### Responses

**Fig. 1:**
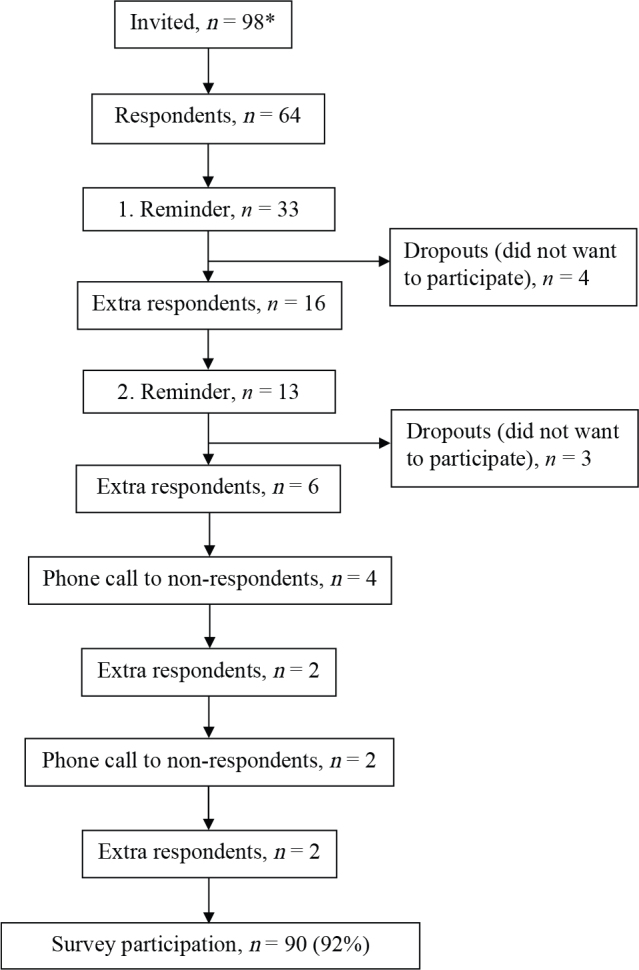
Flowchart illustrating municipality participation in the survey. *There are a total of 98 municipalities in Denmark, but 2 of them are collaborating in the delivery of rehabilitation after discharge from hospital.

The participation rate was 92% (90/98 municipalities) (Fig. 1) across Danish municipalities representing a total of 5.35 million inhabitants in Denmark (Table I). About half of the municipalities reported the use of a standardized screening tool, e.g., 6-minute walk test (6MWT) (21), the New Mobility Score (NMS) (22) (or similar) for the stratification of their provided rehabilitation. By stratification, it is meant that the screening tool can be used to plan the exercise programme, for example, based on functional ability. Only 4% (4/90) of the municipalities used a standardized screening tool for cognitive impairment at the beginning of the rehabilitation. Forty-eight% reported that they had a formal description of the rehabilitation provided to patients with HF, while only 13% had a description of the rehabilitation offered for patients with HF-SCI. In addition, 13% had a specific course provided for the caregivers of patients with HF-SCI.

**Table I:** Oversight of the responding municipalities in the survey

Responding municipalities*	90/98 = 92%
Percentage of responding municipalities from the 5 regions in Denmark:	
- Region Zealand (0.85 million inhabitants)	16/17 = 94%
- Capital Region (1.892 million inhabitants)	26/28 = 93%
- Region of Southern Denmark (1.24 million inhabitants)	19/22 = 86%
- Central Region Denmark (1.36 million inhabitants)	18/19 = 95%
- North Denmark Region (0.60 million inhabitants)	11/11 = 100%
Total number of inhabitants in Denmark 2023	5.94 million

* There are 98 municipalities in Denmark but 2 of them are collaborating in the delivery of rehabilitation after discharge from hospital.

Across all settings and for those with HF or with HF-SCI, more than 74% of the respondents answered that they had the option to allocate extended rehabilitation beyond the standard practice when necessary. In most cases, it was reported that physiotherapists were responsible for the delivering of the physical rehabilitation and across settings and levels of cognition. However, for HF-SCI at 24-H, it was reported by 61% that both physiotherapists and occupational therapists were primarily responsible for the physical rehabilitation (Table SII).

### The delivery of sessions across settings and levels of cognition

The reported number of sessions delivered was typically 1–2 times weekly except at the 24-H setting where sessions were provided more frequently (> 3 times/week) (Table II, Table SII). There was a consistent pattern in the reported number of delivery of sessions across both groups.

**Table II:** Reported rehabilitation offered per week to patients with hip fracture (HF) and with HF and signs of cognitive impairment (HF-SCI)

Municipality setting	Group, *n*	1–2 times, *n* (%)	3–4 times, *n* (%)	All weekdays, *n* (%)	*p-*value
Outpatient healthcare centres	HF, *n* = 90	90 (100)	0 (0)	0 (0)	Not appropriate
	HF-SCI, *n* = 89	87 (97.8)	2 (2.2)	0 (0)	
Home-based care	HF, *n* = 90	85 (94.4)	5 (5.6)	0 (0)	0.36^a^
	HF-SCI, *n* = 90	83 (92.2)	5 (5.6)	2 (2.2)	
24-h care facilities	HF, *n* = 89	29 (32.6)	36 (40.4)	24 (27.0)	0.85^b^
	HF-SCI, *n* = 89	29 (32.6)	33 (37.1)	27 (30.3)	
Nursing home facilities	HF, *n* = 89	75 (84.3)	7 (7.9)	7 (7.9)	0.93^b^
	HF-SCI, *n* = 89	75 (84.3)	8 (9.0)	6 (6.7)	

Data are expressed as numbers (*n*) in percentage (%) of municipalities responding.

a = Fisher’s exact test,

b = Pearson χ^2^ test. *p*-values represent differences between HF and HF-SCI for the number of sessions offered.

The longest duration per session in minutes was reported as indicated on the OPC (46–60 min), for HB sessions as 31–45 min, while sessions mostly for 24-H and NHF were reported to last between 0 and 45 min. No difference was seen between the HF and HF-SCI groups (Table III).

**Table III:** Reported rehabilitation offered per session (in minutes) to patients with hip fracture (HF) and with HF and signs of cognitive impairment (HF-SCI)

Municipality setting	Group, *n*	0–30 min.	31–45 min.	46–60 min.	*p*-value
Outpatient healthcare centres	HF, *n* = 90	2 (2.2)	23 (25.6)	65 (72.2)	0.43^a^
	HF-SCI, *n* = 89	4 (4.5)	28 (31.5)	57 (64.0)	
Home-based care	HF, *n* = 90	13 (14.4)	56 (62.2)	21 (23.3)	0.81^b^
	HF-SCI, *n* = 90	16 (17.8)	55 (61.1)	19 (21.1)	
24-h care facilities	HF, *n* = 89	22 (24.7)	52 (58.4)	15 (16.9)	0.13^b^
	HF-SCI, *n* = 89	33 (37.1)	47 (52.8)	9 (10.1)	
Nursing home facilities	HF, *n* = 89	38 (42.7)	44 (49.4)	7 (7.9)	0.47^b^
	HF-SCI, *n* = 89	37 (41.6)	40 (44.9)	12 (13.5)	

Data are expressed as numbers (*n*) in percentage (%) of municipalities responding.

a = Fisher’s exact test,

b = Pearson χ^2^ test. *p*-values represent differences between HF and HF-SCI for the duration of sessions.

In general, the exercise programmes across settings lasted 5–12 weeks, apart from 24-H, where programmes typically had a shorter duration of 0–8 weeks (Table IV, Table SII). Furthermore, it was found that patients with HF had shorter programmes at 24-H compared with those with HF-SCI (Table IV).

**Table IV:** Reported rehabilitation offered in weeks to patients with hip fracture (HF) and with HF and signs of cognitive impairment (HF-SCI)

Municipality setting	Group, *n*	0–4 weeks, *n* (%)	5–8 weeks, *n* (%)	9–12 weeks, *n* (%)	+13 weeks, *n* (%)	*p*-value
Outpatient healthcare centres	HF, *n* = 90	0 (0)	36 (40.0)	46 (51.1)	8 (8.9)	0.66^a^
	HF-SCI, *n* = 89	1 (1.1)	39 (43.8)	40 (44.9)	9 (10.1)	
Home-based care	HF, *n* = 90	12 (13.3)	47 (52.2)	26 (28.9)	5 (5.6)	0.36^b^
	HF-SCI, *n* = 90	5 (5.6)	51 (56.7)	29 (32.2)	5 (5.6)	
24-h care facilities	HF, *n* = 89	50 (56.2)	27 (30.3)	9 (10.1)	3 (3.4)	0.03^a^
	HF-SCI, *n* = 89	34 (38.2)	29 (32.6)	23 (25.8)	3 (3.4)	
Nursing home facilities	HF, *n* = 89	6 (6.7)	45 (50.6)	32 (36.0)	6 (6.7)	0.99^b^
	HF-SCI, *n* = 89	5 (5.6)	45 (50.6)	33 (37.1)	6 (6.7)	

Data are expressed as numbers (*n*) in percentage (%) of municipalities responding.

a = Fisher’s exact test,

b = Pearson χ^2^ test. *p*-values represent differences between HF and HF-SCI for the timeframe of rehabilitation offered.

### Exercise modalities delivered across settings and levels of cognition.

The 3 most reported exercise modalities across all settings for patients with HF or HF-SCI were skill/functional tasks, strengthening exercises, and balance. Many respondents had indicated that these 3 modalities were more often used for older adults with HF than with HF-SCI, except at OPC (Table V).

**Table V:** Reported rehabilitation modalities offered to patients with hip fracture (HF) and with HF and signs of cognitive impairment (HF-SCI) at 4 different municipality sites

Municipality setting	Outpatient healthcare centres			Home-based care			24-h care facilities			Nursing home facilities		
Group, *n*	HF, *n* = 90	HF-SCI, *n* = 90	*p*-value	HF, *n* = 90	HF-SCI, *n* = 90	*p*-value	HF, *n* = 89	HF-SCI, *n* = 89	*p*-value	HF, *n* = 89	HF-SCI, *n* = 89	*p-*value
Warm-up	88 (97.8)	80 (88.9)	0.02^a^	72 (80.0)	32 (35.6)	< 0.001^b^	71 (79.8)	58 (64.4)	0.03^b^	46 (51.1)	36 (40.0)	0.13^b^
Cardio	25 (27.8)	13 (14.4)	0.03^b^	8 (9)	5 (5.6)	0.38^a^	8 (9.0)	7 (7.8)	0.79^b^	5 (5.6)	1 (1.1)	0.10^a^
Fitness	31 (34.4)	36 (40.0)	< 0.44^b^	27 (30.0)	19 (21.1)	0.17^b^	26 (29.2)	24 (26.7)	0.74^b^	15 (16.7)	18 (20.0)	0.56^b^
Strength exercise	70 (77.8)	49 (54.4)	< 0.001^b^	24 (26.7)	15 (16.7)	0.10^b^	29 (32.6)	18 (20.0)	< 0.001^b^	18 (20.0)	15 (16.7)	0.56^b^
Strengthening exercises^c^	60 (66.7)	70 (77.8)	0.06^a^	76 (84.4)	72 (80.0)	0.88^b^	74 (83.1)	72 (80.0)	0.87^b^	71 (79.8)	66 (73.3)	0.23^b^
Balance	87 (96.7)	81 (90.0)	0.07^a^	61 (67.8)	44 (48.9)	0.01^b^	66 (74.2)	56 (62.2)	0.11^b^	44 (48.9)	43 (47.8)	0.88^b^
Skill/Functional tasks	81 (90.0)	78 (86.7)	0.49^b^	90 (100.0)	87 (96.7)	0.08^a^	87 (97.8)	86 (95.6)	0.65^a^	86 (95.6)	85 (94.4)	0.70^a^
Activities of daily living (ADL)	4 (4.4)	11 (12.2)	0.06^a^	52 (57.8)	53 (58.9)	0.88^b^	63 (70.8)	64 (71.1)	0.87^b^	43 (47.8)	51 (56.7)	0.23^b^
Outdoor walking	42 (46.7)	42 (46.7)	1.00^b^	71 (78.9)	60 (66.7)	0.07^b^	36 (40.4)	26 (28.9)	0.12^b^	29 (32.2)	28 (31.1)	0.87^b^
Other	5 (5.6)	1 (1.1)	0.1^a^	9 (10.0)	7 (7.8)	0.60^b^	7 (7.9)	4 (4.4)	0.35^a^	5 (5.6)	4 (4.4)	0.73^a^

Data are expressed as numbers (*n*) in percentage (%) of municipalities responding. n/a = none available,

a = Fisher’s exact test,

b = Pearson χ^2^ test. *p*-values represent differences between HF and HF-SCI for the given modality and setting,

c = strengthening exercises defined as more than +15 repetitions per exercise.

Overall, the types of modalities delivered were similar across all settings. However, a higher number of municipalities reported that individuals with HF more frequently received warm-up sessions at OPC, HB, and 24-H; additionally, cardio exercise was more often reported for HF than HF-SCI at OPC, and strength exercises were more commonly provided to HF than HF-SCI at both OPC and 24-H (Table V).

### Use of outcome measures across settings and levels of cognition

**Fig. 2:**
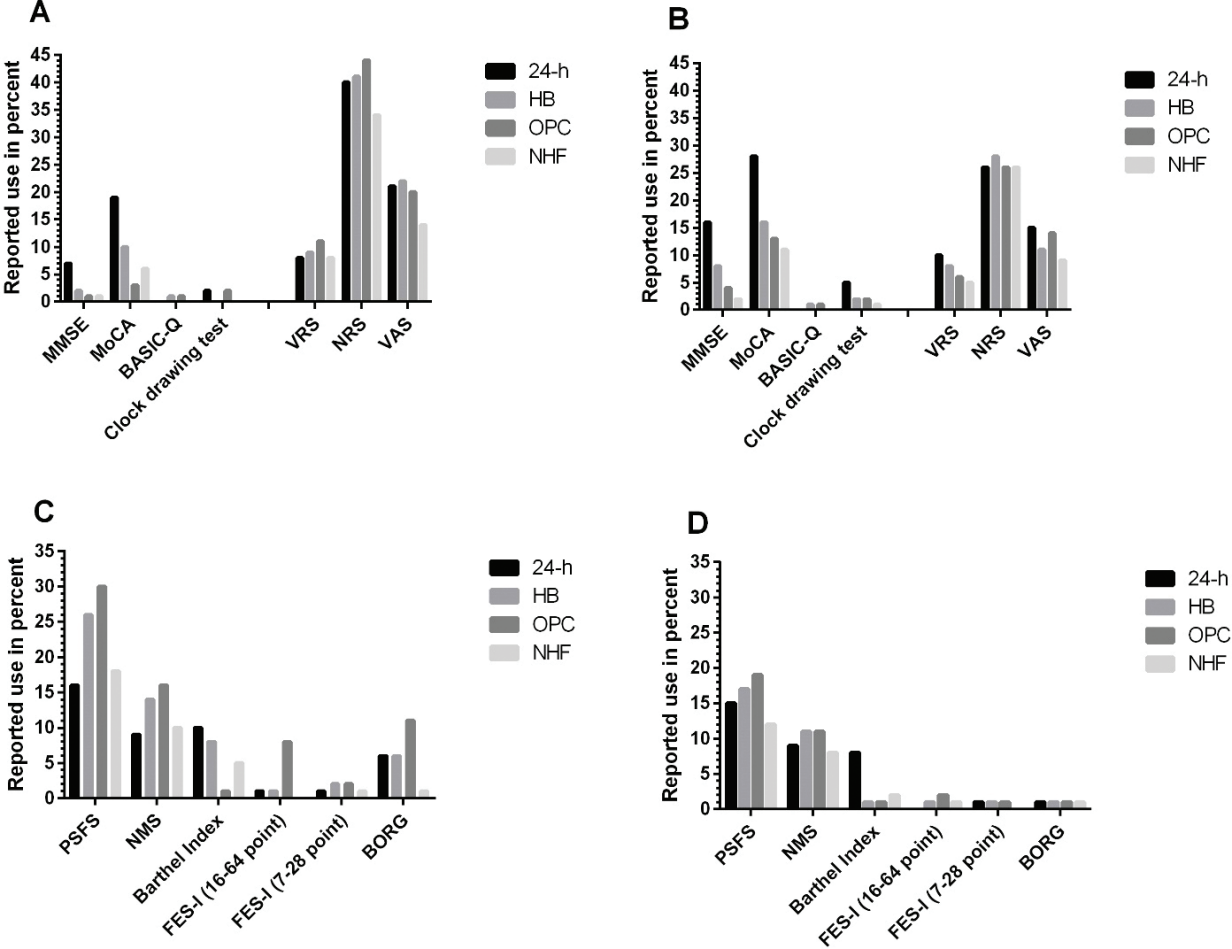
Reported use of patient-reported outcome measures (PROMs, in percentages) of cognition, pain, and activity distributed in 4 municipality-based locations and level of cognition: 2A, 2C = patients with hip fracture who are cognitively intact, 2B, 2D = patients with hip fracture and signs of cognitive impairment. 24-h = 24-hour care facilities, HB = home-based care, OPC = outpatient healthcare centres, NHF = nursing home facilities, MMSE = Mini Mental State Examination, MoCA = Montreal Cognitive Assessment, BASIC-Q = Brief Assessment of Impaired Cognition Questionnaire, VRS = Verbal Rating Scale, NRS = Numeric Rating Scale, VAS = Visual Analogue Scale, PSFS = Patient Specific Functional Scale, NMS = New Mobility Score, FES-I (16–64 point) = Falls Efficacy Scale – International (long: 16–64 points), FES-I (7–28 points) = Fall Efficacy Scale – International (short: 7–28 points), BORG = Borg Rating of Perceived Exertion.

*PROMs*. A PROM for cognition was not reported as widely used in the municipality settings but therapists had a slight preference for the Montreal Cognitive Assessment (MoCA) test at 24-H (Fig. 2A, 2B), otherwise the use of PROM cognition tests was sparse. In the pain assessments (PROM pain) the Numeric Rating Scale (NRS) was the test most often chosen. The reported preference in PROM activity was the Patient Specific Functional Scale (PSFS), followed by the NMS, with almost no use of the 2 “fear of falling” measures: Falls Efficacy Scale-International (FES-I) long and short version (Fig. 2C, 2D), Table SIII).

**Fig. 3:**
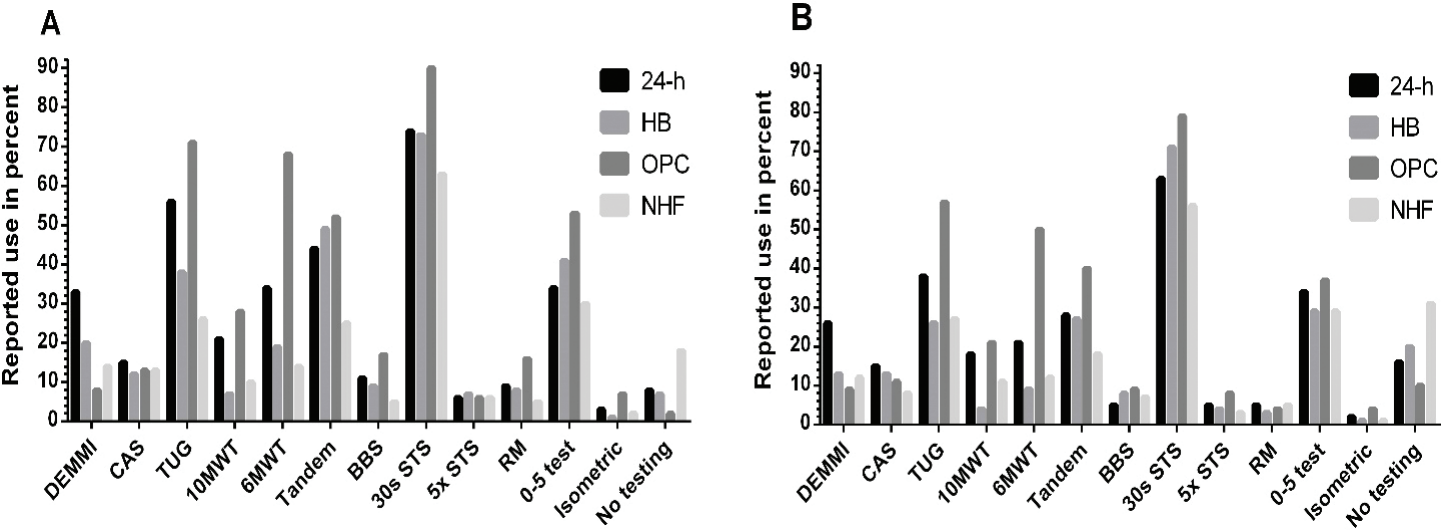
Reported use of performance-based outcome measures in percentages distributed in 4 municipality-based locations and the level of cognition: 3A = patients with hip fracture who are cognitively intact, 3B = patients with hip fracture and signs of cognitive impairment. 24-h = 24-hour care facilities, HB = home-based care, OPC = outpatient healthcare centres, NHF = nursing home facilities, DEMMI = De Morton Mobility Index, CAS = Cumulated Ambulation Score, TUG = Timed Up & Go test, 10MWT = 10-Meter Walking Test, 6MWT = 6-Minute Walk Test, Tandem = Short Physical Performance Test, balance element test, BBS = Berg’s Balance Scale, 30s STS = 30 second Sit-To-Stand test, 5x STS = Five Times Sit-To-Stand test, RM = Repetition Maximum, 0–5 Test = 0–5 muscle grading test, Isometric = isometric muscle testing.

*Performance-based measures*. The 30 s Sit-To-Stand test (30 s STS), the Timed Up&Go (TUG) test, the Short Physical Performance Test – balance element (Tandem), and the 0–5 muscle grading test (0–5 muscle testing) were reported as the most used outcome measure across all settings (Fig. 3A, 3B, Table SIII). In addition, the 6MWT was also used in more than 30% at OPC and (HF and HF-SCI) and 24-H (HF) (Fig. 3A, 3B, Table SIII). No testing was used in more than 30% of the cases at NHF (HF-SCI, Fig. 3B).

## DISCUSSION

This national E-survey had a 92% response rate and found that physiotherapists were most commonly responsible for delivering exercises and assessments, and that the rehabilitation services offered were largely similar for both HF and HF-SCI groups, with few exceptions. PROMs were not widely used in clinical practice, whereas performance-based tests were more commonly applied.

More than a decade ago, Kronborg et al. (15) conducted a similar survey covering over 50% of Danish municipalities. At that time, only approximately half of the responding municipalities reported having formal rehabilitation protocols or programme descriptions – findings that remain largely consistent with our current study, despite broader national coverage. This persistent lack of structured descriptions is striking, given the substantial growth in randomized trials and clinical practice guidelines in recent years, which have established a solid evidence base for rehabilitation in patient post-fracture (3).

Notably, our study also shows that only 13% of municipalities report having a formal programme specifically tailored to patients with HF-SCI, and just 4% use cognitive screening tools in municipal rehabilitation settings. Evidence from an Australian/New Zealand registry study including > 49,000 patients with HF demonstrated that patients with HF-SCI received markedly poorer care compared with their cognitively intact counterparts, particularly in areas such as pain assessment, mobilization, and overall rehabilitation services (11). This lack of attention to a large and fragile subgroup of patients with HF may reflect a persistent knowledge or implementation gap in the field. A Canadian survey (23) among 130 physiotherapists working with people living with dementia revealed educational needs for dementia knowledge, and strategies to manage behavioural and cognitive symptoms. The latter study also recommended that targeted education for physiotherapists should be included in entry-to-practice training and be available for those at a postgraduate level. This apparent knowledge gap in the rehabilitation of patients with HF-SCI may contribute to the prevailing perception that this subgroup lacks rehabilitative potential (10). At the same time, it also reflects structural and economic constraints that limit the implementation of more individualized and person-centred approaches – an element widely recognized as essential for effective rehabilitation outcomes in people living with dementia (13). Although our findings indicate that, with few exceptions, the reported content of rehabilitation services is similar for individuals with HF and HF-SCI, this conclusion relies on self-reported practices, which may not accurately represent actual care delivered. While the apparent lack of exclusion criteria for those with HF-SCI may indicate inclusive intent, the absence of guidance on how to tailor rehabilitation for this subgroup highlights the need for more specific recommendations.

### Exercise modalities

Largely consistent with current evidence and recommendations (3, 24, 25), we found that the 3 most frequently reported types across all settings for patients with HF or HF-SCI were skills/functional tasks, strengthening, and balance exercises. A systematic review and meta-analysis from Hulsbæk et al. (24) reported low-quality evidence indicating a small-to-moderate effect of exercise therapy targeting activities of daily living (ADL), lower limb muscle strength, and balance. Correspondingly, a Cochrane Review by Fairhall et al. (25) demonstrated that interventions incorporating gait, balance, and skills/functional tasks were particularly effective in improving mobility following hip fracture. Furthermore, the American Clinical Practice Guideline (CPG) regarding physical therapy management of patients with HF (3) recommends that exercise modalities in the post-acute phase – delivered in home care and community settings – should include gait exercise, skills/functional tasks, balance, and high-intensity strength exercise.

However, we did find that all exercise modalities were more frequently reported as being used for patients with HF compared with those with HF-SCI, with strength exercises more often applied at OPC and 24-H settings for the HF group. This discrepancy warrants attention in clinical practice, especially given that the American CPG explicitly recommends providing physical therapy using similar interventions for individuals with and without dementia (3). This suggests a potential to enhance and standardize rehabilitation offerings for patients with HF-SCI in addition to what is currently reported in Danish municipalities. Policymakers and managers should place greater emphasis on, for example, conducting systematic medical record audits to ensure that patients with HF-SCI receive standardized rehabilitation aligned with that offered to patients with HF, thereby maintaining a high professional standard in the delivery of services.

Beyond the standardization of post-discharge rehabilitation for patients with HF, particularly those with HF-SCI, it is also relevant to consider how rehabilitation is organized in other countries. While post-discharge rehabilitation in Denmark primarily relies on physiotherapists as gatekeepers in collaboration with general practitioners, studies from Sweden (7) and Taiwan (26) describe the use of multidisciplinary teams involving multiple professional disciplines to address the complex challenges of post-discharge rehabilitation and potentially improve rehabilitation pathways.

### Outcome measures

Detailed information was gathered throughout the use of 25 commonly used outcome measures in the different municipality settings. Fewer than one-third of respondents reported using outcome measures for cognition, which is concerning given that up to 40% of patients with HF may present with cognitive impairment (5, 6). The identification of such impairment could be important for guiding appropriate care strategies and tailoring the patient’s recovery pathway, and might also help to direct the care provided or determine the most suitable rehabilitation trajectory. While it is not the role of physiotherapists to diagnose cognitive disorders, screening can serve to confirm clinical observations that are valuable for tailoring rehabilitation plans. Physiotherapists can inform the patient about the result of the cognitive screening, so the patient can contact their general practitioner if needed. If the patient is unable to do so and has given consent, the physiotherapist can also contact the general practitioner directly.

### PROM pain

Surprisingly, given the high prevalence of HF-related pain in this patient group (27), we found that only 4 out of 10 respondents reported using pain assessment scales in practice. Furthermore, the NRS and Visual Analogue Scale (VAS) were reported as more commonly used than the reliable (28), recommended (3), and clinically applicable Verbal Rating Scale (VRS) (29). Inadequate pain assessment and effective pain management may contribute to uncertainty among patients with HF regarding the safety and appropriateness of engaging in rehabilitation activities (30).

### PROM activity

It was generally reported that the use of PROMs tools was limited. Fewer than 1 in 3 reported the use of the PSFS, the NMS less than 16%, and the FES-I under 10%. Additionally, it is worth noting that the PSFS was the most used, despite lacking evidence of reliability and validity in the HF population and not being recommended by the American CPG (3).

It was also striking that the use of the NMS and the FES-I was underrepresented in clinical practice, despite being recommended (3) with good reliability (22, 31) and validity (31,32). The NMS can be used to identify the patient’s pre-fracture functional level and thus serve as a tool for setting rehabilitation goals, specifically aiming for the patient to regain their previous level of function. The NMS is suggested as one of the core outcome measures for patients with HF (33), in addition to being employed in both the Danish and Irish HF registries. As most HF occur because of a fall, it is important for clinicians to assess the patient’s fear of falling, for which the FES-I can be used. Evidence shows that fear of falling is prevalent among older adults with HF and is significantly associated with physical function and anxiety levels and important for patients with HF (34–36). If the clinician fails to identify the patient’s fear of falling, this may act as a barrier to the rehabilitation process, potentially resulting in an unnecessarily prolonged recovery.

### Performance-based scores/outcomes

Respondents indicated that the 30 s STS, TUG, and Tandem tests were the 3 most used performance-based measures across levels of cognition and care settings. Within the population of patients with HF, to our knowledge there is no evidence supporting the use of the 30 s STS test, while acceptable reliability and validity have been established for both the TUG and Tandem tests, which are also recommended by the American CPG (3). In post-discharge municipal rehabilitation, there is often a strong focus on regaining pre-fracture levels of function. Baseline and follow-up assessments are commonly conducted, typically targeting activities such as walking, sit-to-stand transitions, or balance. In this context, clinicians should prioritize the use of outcome measures that have been thoroughly validated for the specific population, including those supported by reliability studies that establish measurement error – ensuring that observed improvements in performance-based measures reflect true change beyond measurement variability.

### Strength and limitations

One major strength of this study is the high response rate from 92% of all municipalities in Denmark, which provides a highly representative picture of what rehabilitation is being offered in the country’s municipalities.

Second, we extended previous research by asking the municipalities about the services they provide not only to individuals with HF but also to those with HF-SCI. In addition, we inquired about the services provided across the 4 typical settings where exercise interventions are delivered to older adults with HF and HF-SCI. This provides a greater level of detail than previously published regarding what is being offered and where.

A key limitation of this survey is its reliance on self-reported data from municipal representatives, which likely reflects intended rather than actual service delivery; without patient-level or observational data, discrepancies between reported and delivered care for patients with HF and HF-SCI cannot be ruled out. Moreover, the extent to which these findings are transferable to healthcare systems beyond Denmark remains unclear.

### Conclusion

The survey, covering more than 92% of all Danish municipalities, found that they generally provide similar inclusive services to patients with HF regardless of cognitive status. However, specific adaptations to meet the needs of patients with HF-SCI appear to be lacking. Municipalities may use the results of this study to benchmark their services against national trends, thereby supporting more structured planning of post-discharge rehabilitation for patients with HF, including those with HF-SCI. Future research should explore international post-discharge rehabilitation practices to examine how these services differ or are similar across countries. Moreover, the current findings from Denmark should be validated by investigating what patients are actually offered in practice.

## Supplementary Material




