# Supplementary material for: EXERCISE MODALITIES AND OUTCOME MEASURES USED IN OLDER ADULTS AFTER HIP FRACTURE WITH OR WITHOUT SIGNS OF COGNITIVE IMPAIRMENT: A NATIONAL CROSS-SECTIONAL E-SURVEY OF 90 OUT OF 98 MUNICIPALITIES IN DENMARK
Source: J Rehabil Med. 2026 Feb 11;58:44207. doi: 10.2340/jrm.v58.44207 (PMC12914642; doi:10.2340/jrm.v58.44207)
Supplement: Supplementary file 1 [file JRM-58-44207-s1.pdf]

Supplementary material has been published as submitted. It has not been copyedited, or typeset by Journal of Rehabilitation Medicine

Appendix S1 - Questionnaire in English:

Exercise modalities and outcome measures used in older adults after hip fracture with or without signs of cognitive impairment. A national cross-sectional E-survey

Part One: Preliminary questions

1. Which municipality do you work in?	

2. Are there any of your patients with hip fracture who do not receive rehabilitation in the municipality but elsewhere? (e.g., with a private provider, in collaboration with other municipalities, etc.)	
YES	
NO	

3. Do you use any form of assessment tool/screening instrument for stratifying and organising your rehabilitation efforts for patients with hip fracture?	
YES	
NO	
If YES, please specify which assessment tools/screening instruments you use in your municipality.	

4. Are patients with hip fracture screened with a standardised test for signs of cognitive impairment prior to the start of rehabilitation in your municipality?	
YES	
NO	
If yes, please describe which cognitive screening tool(s) you typically use in your municipality (Select one or more of the answers that apply to your setting).	
Mini-Mental State Examination (MMSE)	
Montreal Cognitive Assessment (MoCA)	
Brief Assessment of Impaired Cognition Questionaire (BASIC-Q)	
Clock Drawing Test	
Nothing	
Other (describe)	

Part Two: Questions regarding rehabilitation and testing of patients with hip fracture residing in 24-hour care facilities.

5. Who primarily conducts the rehabilitation of patients with hip fracture who receive exercise in a 24-hour care facility? (Mark the relevant category)	
Physiotherapist	
Occupational Therapist	
Both Physiotherapist and Occupational Therapist	
Physiotherapy Assistant	
Other	

6. How often do patients with hip fracture exercise, who receive rehabilitation in a 24-hour care facility? (Mark the relevant category)	
Fewer than 1 session/week	
1-2 sessions/week	
3-4 sessions/week	
Daily	
Several times daily	

7. What is the typical overall time frame (in minutes) for a single exercise session for patients with hip fracture receiving rehabilitation in a 24-hour care facility? (Mark the time interval that indicates the maximum duration of a session)	
0-15 min.	
16-30 min.	
31-45 min.	
46-60 min.	
61-75 min.	
+76 min.	

8. What is the typical overall time frame (in weeks) for the total period of rehabilitation for patients with hip fracture receiving rehabilitation in a 24-hour care facility? (Mark the week interval that typically indicates the total number of exercise weeks)	
0-2 weeks	
3-4 weeks	
5-6 weeks	
7-8 weeks	

9-10 weeks	
11-12 weeks	
13+ weeks	

9. Is the rehabilitation period at the 24-hour care facility time-limited but extendable if needed/based on the goals for the patients with hip fracture?	
YES	
NO	

10. How are the rehabilitation sessions conducted at the 24-hour care facility for patients with hip fracture?	
Group-based/classes	
Individual (one-on-one)	
Both options	

11. What modalities of exercise does a typical session consist of at a 24-hour care facility for patients with hip fracture? (Mark the category/categories that are relevant)	
Warm-up (e.g. on a stationary bike, walking with rollator))	
Cardiovascular exercise (BORG intensity level 14-15)	
Fitness (below BORG 14 intensity)	
Strength exercises	
Strengthening exercises (defined as more than +15 repetitions per exercise) (with weightcuffs, strength training machines, free weights, etc)	
Balance exercises (static or dynamic)	
Skill/functional tasks (flights, in/out of bed, up from floor)	
Exercises of relevant activities of daily living (ADL)(bathing, getting dressed, eat/drinking)	
Outdoor walking (with or without walking aid)	
Other	

12. Which tests do you use for patients with hip fracture who receive their rehabilitation at a 24-hour care facility? (Mark the relevant test(s))	
Mini-Mental State Examination (MMSE)	
Montreal Cognitive Assessment (MoCA)	
Brief Assessment of Impaired Cognition Questionaire (BASIC-Q)	
Clock Drawing Test	
Patient Specific Functional Scale (PSFS)	

De Morton Mobility Index (DEMMI)	
Cumulated Ambulation Score (CAS)	
New Mobility Score (NMS)	
Timed Up&Go (TUG)	
10-Meter Walking Test	
6-Minute Walk Test	
Verbal Rating Scale (VRS)	
Numeric Rang Scale (NRS)	
Visual Analog Scale (VAS)	
Borg Rating of Perceived Exertion	
Tandem test (balance)	
Berg´s Balance Scale	
Falls Efficacy Scale - International (FES-I) (16-64 point scale)	
Short Falls Efficacy Scale - International (sFES-I) (7-28 point scale)	
30-second Sit-To-Stand test	
Five Times Sit-To-Stand test	
Barthel Index	
Repetition Maximum (RM)	
0-5 muscle grading test	
Isometric muscle testing (e.g. with a dynamometer)	
Nothing	

Rehabilitation and testing of patients with hip fracture in home-based care.

13. Who primarily conducts the rehabilitation of patients with hip fracture who receive exercise in home-based care? (Mark the relevant category)	
Physiotherapist	
Occupational Therapist	
Both Physiotherapist and Occupational Therapist	
Physiotherapy Assistant	
Other	

14. How often do patients with hip fracture exercise, who receive rehabilitation in home-based care? (Mark the relevant category)	
Fewer than 1 session/week	
1-2 sessions/week	

3-4 sessions/week	
Daily	
Several times daily	

15. What is the typical overall time frame (in minutes) for a single exercise session for patients with hip fracture receiving rehabilitation in home-based care? (Mark the time interval that indicates the maximum duration of a session)	
0-15 min.	
16-30 min.	
31-45 min.	
46-60 min.	
61-75 min.	
+76 min.	

16. What is the typical overall time frame (in weeks) for the total period of rehabilitation for patients with hip fracture receiving rehabilitation in home-based care? (Mark the week interval that typically indicates the total number of exercise weeks)	
0-2 weeks	
3-4 weeks	
5-6 weeks	
7-8 weeks	
9-10 weeks	
11-12 weeks	
13+ weeks	

17. Is the rehabilitation period in the home-based care time-limited but extendable if needed/based on the goals for the patients with hip fracture?	
YES	
NO	

18. How are the rehabilitation sessions conducted at the home-based care for patients with hip fracture?	
Group-based/classes	
Individual (one-on-one)	
Both options	

19. What modalities of exercise does a typical session consist of as home-based care for patients with hip fracture? (Mark the category/categories that are relevant)	
Warm-up (e.g. on a stationary bike, walking with rollator))	
Cardiovascular exercise (BORG intensity level 14-15)	
Fitness (below BORG 14 intensity)	
Strength exercises	
Strengthening exercises (defined as more than +15 repetitions per exercise) (with weightcuffs, strength training machines, free weights, etc)	
Balance exercises (static or dynamic)	
Skill/functional tasks (flights, in/out of bed, up from floor)	
Exercises of relevant activities of daily living (ADL)(bathing, getting dressed, eat/drinking)	
Outdoor walking (with or without walking aid)	
Other	

20. Which tests do you use for patients with hip fracture who receive their rehabilitation as home-based care? (Mark the relevant test(s))	
Mini-Mental State Examination (MMSE)	
Montreal Cognitive Assessment (MoCA)	
Brief Assessment of Impaired Cognition Questionaire (BASIC-Q)	
Clock Drawing Test	
Patient Specific Functional Scale (PSFS)	
De Morton Mobility Index (DEMMI)	
Cumulated Ambulation Score (CAS)	
New Mobility Score (NMS)	
Timed Up&Go (TUG)	
10-Meter Walking Test	
6-Minute Walk Test	
Verbal Rating Scale (VRS)	
Numeric Rang Scale (NRS)	
Visual Analog Scale (VAS)	
Borg Rating of Perceived Exertion	
Tandem test (balance)	
Berg´s Balance Scale	
Falls Efficacy Scale - International (FES-I) (16-64 point scale)	
Short Falls Efficacy Scale - International (sFES-I) (7-28 point scale)	
30-second Sit-To-Stand test	

Five Times Sit-To-Stand test	
Barthel Index	
Repetition Maximum (RM)	
0-5 muscle grading test	
Isometric muscle testing (e.g. with a dynamometer)	
Nothing	

Rehabilitation and testing of patients with hip fracture at outpatient healthcare centers.

21. Who primarily conducts the rehabilitation of patients with hip fracture who receive exercise in an outpatient healthcare center? (Mark the relevant category)	
Physiotherapist	
Occupational Therapist	
Both Physiotherapist and Occupational Therapist	
Physiotherapy Assistant	
Other	

22. How often do patients with hip fracture exercise, who receive rehabilitation in an outpatient healthcare center? (Mark the relevant category)	
Fewer than 1 session/week	
1-2 sessions/week	
3-4 sessions/week	
Daily	
Several times daily	

23. What is the typical overall time frame (in minutes) for a single exercise session for patients with hip fracture receiving rehabilitation in an outpatient healthcare center? (Mark the time interval that indicates the maximum duration of a session)	
0-15 min.	
16-30 min.	
31-45 min.	
46-60 min.	
61-75 min.	
+76 min.	

24. What is the typical overall time frame (in weeks) for the total period of rehabilitation for patients with hip fracture receiving rehabilitation in an outpatient healthcare center? (Mark the week interval that typically indicates the total number of exercise weeks)	
0-2 weeks	
3-4 weeks	
5-6 weeks	
7-8 weeks	
9-10 weeks	
11-12 weeks	
13+ weeks	

25. Is the rehabilitation period at the outpatient healthcare center time-limited but extendable if needed/based on the goals for the patients with hip fracture?	
YES	
NO	

26. How are the rehabilitation sessions conducted at an outpatient healthcare center for patients with hip fracture?	
Group-based/classes	
Individual (one-on-one)	
Both options	

27. What modalities of exercise does a typical session consist of at an outpatient healthcare center for patients with hip fracture? (Mark the category/categories that are relevant)	
Warm-up (e.g. on a stationary bike, walking with rollator))	
Cardiovascular exercise (BORG intensity level 14-15)	
Fitness (below BORG 14 intensity)	
Strength exercises	
Strengthening exercises (defined as more than +15 repetitions per exercise) (with weightcuffs, strength training machines, free weights, etc)	
Balance exercises (static or dynamic)	
Skill/functional tasks (flights, in/out of bed, up from floor)	
Exercises of relevant activities of daily living (ADL)(bathing, getting dressed, eat/drinking)	
Outdoor walking (with or without walking aid)	
Other	

28. Which tests do you use for patients with hip fracture who receive their rehabilitation at an outpatient healthcare center? (Mark the relevant test(s))	
Mini-Mental State Examination (MMSE)	
Montreal Cognitive Assessment (MoCA)	
Brief Assessment of Impaired Cognition Questionaire (BASIC-Q)	
Clock Drawing Test	
Patient Specific Functional Scale (PSFS)	
De Morton Mobility Index (DEMMI)	
Cumulated Ambulation Score (CAS)	
New Mobility Score (NMS)	
Timed Up&Go (TUG)	
10-Meter Walking Test	
6-Minute Walk Test	
Verbal Rating Scale (VRS)	
Numeric Rang Scale (NRS)	
Visual Analog Scale (VAS)	
Borg Rating of Perceived Exertion	
Tandem test (balance)	
Berg´s Balance Scale	
Falls Efficacy Scale - International (FES-I) (16-64 point scale)	
Short Falls Efficacy Scale - International (sFES-I) (7-28 point scale)	
30-second Sit-To-Stand test	
Five Times Sit-To-Stand test	
Barthel Index	
Repetition Maximum (RM)	
0-5 muscle grading test	
Isometric muscle testing (e.g. with a dynamometer)	
Nothing	

Rehabilitation and testing of patients with hip fracture residing in nursing home facilities.

29. Who primarily conducts the rehabilitation of patients with hip fracture who receive exercise in a nursing home facility? (Mark the relevant category)	
Physiotherapist	
Occupational Therapist	
Both Physiotherapist and Occupational Therapist	

Physiotherapy Assistant	
Other	

30. How often do patients with hip fracture exercise, who receive rehabilitation in a nursing home facility? (Mark the relevant category)	
Fewer than 1 session/week	
1-2 sessions/week	
3-4 sessions/week	
Daily	
Several times daily	
31. What is the typical overall time frame (in minutes) for a single exercise session for patients with hip fracture receiving rehabilitation in a nursing home facility? (Mark the time interval that indicates the maximum duration of a session)	
0-15 min.	
16-30 min.	
31-45 min.	
46-60 min.	
61-75 min.	
+76 min.	

32. What is the typical overall time frame (in weeks) for the total period of rehabilitation for patients with hip fracture receiving rehabilitation in a nursing home facility? (Mark the week interval that typically indicates the total number of exercise weeks)	
0-2 weeks	
3-4 weeks	
5-6 weeks	
7-8 weeks	
9-10 weeks	
11-12 weeks	
13+ weeks	

33. Is the rehabilitation period at the nursing home facility time-limited but extendable if needed/based on the goals for the patients with hip fracture?

YES	
NO	

34. How are the rehabilitation sessions conducted at the nursing home facility for patients with hip fracture?	
Group-based/classes	
Individual (one-on-one)	
Both options	

35. What modalities of exercise does a typical session consist of in a nursing home facility for patients with hip fracture? (Mark the category/categories that are relevant)	
Warm-up (e.g. on a stationary bike, walking with rollator))	
Cardiovascular exercise (BORG intensity level 14-15)	
Fitness (below BORG 14 intensity)	
Strength exercises	
Strengthening exercises (defined as more than +15 repetitions per exercise) (with weightcuffs, strength training machines, free weights, etc)	
Balance exercises (static or dynamic)	
Skill/functional tasks (flights, in/out of bed, up from floor)	
Exercises of relevant activities of daily living (ADL)(bathing, getting dressed, eat/drinking)	
Outdoor walking (with or without walking aid)	
Other	

36. Which tests do you use for patients with hip fracture who receive their rehabilitation in a nursing home facility? (Mark the relevant test(s))	
Mini-Mental State Examination (MMSE)	
Montreal Cognitive Assessment (MoCA)	
Brief Assessment of Impaired Cognition Questionaire (BASIC-Q)	
Clock Drawing Test	
Patient Specific Functional Scale (PSFS)	
De Morton Mobility Index (DEMMI)	
Cumulated Ambulation Score (CAS)	
New Mobility Score (NMS)	
Timed Up&Go (TUG)	
10-Meter Walking Test	
6-Minute Walk Test	
Verbal Rating Scale (VRS)	
Numeric Rang Scale (NRS)	
Visual Analog Scale (VAS)	

Borg Rating of Perceived Exertion	
Tandem test (balance)	
Berg´s Balance Scale	
Falls Efficacy Scale - International (FES-I) (16-64 point scale)	
Short Falls Efficacy Scale - International (sFES-I) (7-28 point scale)	
30-second Sit-To-Stand test	
Five Times Sit-To-Stand test	
Barthel Index	
Repetition Maximum (RM)	
0-5 muscle grading test	
Isometric muscle testing (e.g. with a dynamometer)	
Nothing	

37. Do you have a formal description of your rehabilitation offer to patients with hip fracture in your municipality?	
YES	
NO	
If YES, please send it to Jan Overgaard via email: jover@lolland.dk	

Part Three: Patients with hip fracture and signs of cognitive impairment or dementia.

Rehabilitation and testing of patients with hip fracture and signs of cognitive impairment residing at 24-hour care facilities.

38. Is there a service/course for relatives of patients with hip fractures and signs of cognitive impairment in your municipality?	
YES	
NO	

39. Who primarily conducts the rehabilitation of patients with hip fracture and signs of cognitive impairment who receive exercise in a 24-hour care facility? (Mark the relevant category)	
Physiotherapist	
Occupational Therapist	
Both Physiotherapist and Occupational Therapist	
Physiotherapy Assistant	
Other	

40. How often do patients with hip fracture and signs of cognitive impairment exercise, who receive rehabilitation in a 24-hour care facility? (Mark the relevant category)	
Fewer than 1 session/week	
1-2 sessions/week	
3-4 sessions/week	
Daily	
Several times daily	

41. What is the typical overall time frame (in minutes) for a single exercise session for patients with hip fracture and signs of cognitive impairment receiving rehabilitation in a 24-hour care facility? (Mark the time interval that indicates the maximum duration of a session)	
0-15 min.	
16-30 min.	
31-45 min.	
46-60 min.	
61-75 min.	
+76 min.	

42. What is the typical overall time frame (in weeks) for the total period of rehabilitation for patients with hip fracture and signs of cognitive impairment receiving rehabilitation in a 24-hour care facility? (Mark the week interval that typically indicates the total number of exercise weeks)	
0-2 weeks	
3-4 weeks	
5-6 weeks	
7-8 weeks	
9-10 weeks	
11-12 weeks	
13+ weeks	

43. Is the rehabilitation period at the 24-hour care facility time-limited but extendable if needed/based on the goals for the patients with hip fracture and signs of cognitive impairment?

YES	
NO	

44. How are the rehabilitation sessions conducted at the temporary residential care/respite care/rehabilitation facility for patients with hip fracture and signs of cognitive impairment?

Group-based/classes	
Individual (one-on-one)	
Both options	

45. What modalities of exercise does a typical session consist of at a 24-hour care facility for patients with hip fracture and signs of cognitive impairment? (Mark the category/categories that are relevant)	
Warm-up (e.g. on a stationary bike, walking with rollator))	
Cardiovascular exercise (BORG intensity level 14-15)	
Fitness (below BORG 14 intensity)	
Strength exercises	
Strengthening exercises (defined as more than +15 repetitions per exercise) (with weightcuffs, strength training machines, free weights, etc)	
Balance exercises (static or dynamic)	
Skill/functional tasks (flights, in/out of bed, up from floor)	
Exercises of relevant activities of daily living (ADL)(bathing, getting dressed, eat/drinking)	
Outdoor walking (with or without walking aid)	
Other	

46. Which tests do you use for patients with hip fracture and signs of cognitive impairment who receive their rehabilitation at a 24-hour care facility? (Mark the relevant test(s))	
Mini-Mental State Examination (MMSE)	
Montreal Cognitive Assessment (MoCA)	
Brief Assessment of Impaired Cognition Questionaire (BASIC-Q)	
Clock Drawing Test	
Patient Specific Functional Scale (PSFS)	
De Morton Mobility Index (DEMMI)	
Cumulated Ambulation Score (CAS)	
New Mobility Score (NMS)	
Timed Up&Go (TUG)	
10-Meter Walking Test	
6-Minute Walk Test	
Verbal Rating Scale (VRS)	
Numeric Rang Scale (NRS)	
Visual Analog Scale (VAS)	
Borg Rating of Perceived Exertion	
Tandem test (balance)	

Berg´s Balance Scale	
Falls Efficacy Scale - International (FES-I) (16-64 point scale)	
Short Falls Efficacy Scale - International (sFES-I) (7-28 point scale)	
30-second Sit-To-Stand test	
Five Times Sit-To-Stand test	
Barthel Index	
Repetition Maximum (RM)	
0-5 muscle grading test	
Isometric muscle testing (e.g. with a dynamometer)	
Nothing	

Rehabilitation and testing of patients with hip fracture and signs of cognitive impairment in home-based care.

47. Who primarily conducts the rehabilitation of patients with hip fracture and signs of cognitive impairment who receive exercise in home-based care? (Mark the relevant category)	
Physiotherapist	
Occupational Therapist	
Both Physiotherapist and Occupational Therapist	
Physiotherapy Assistant	
Other	

48. How often do patients with hip fracture and signs of cognitive impairment exercise, who receive rehabilitation in a home-based care? (Mark the relevant category)	
Fewer than 1 session/week	
1-2 sessions/week	
3-4 sessions/week	
Daily	
Several times daily	

49. What is the typical overall time frame (in minutes) for a single exercise session for patients with hip fracture and signs of cognitive impairment receiving rehabilitation in home-based care? (Mark the time interval that indicates the maximum duration of a session)	
0-15 min.	
16-30 min.	
31-45 min.	

46-60 min.	
61-75 min.	
+76 min.	

50. What is the typical overall time frame (in weeks) for the total period of rehabilitation for patients with hip fracture and signs of cognitive impairment receiving rehabilitation in home-based care? (Mark the week interval that typically indicates the total number of exercise weeks)	
0-2 weeks	
3-4 weeks	
5-6 weeks	
7-8 weeks	
9-10 weeks	
11-12 weeks	
13+ weeks	

51. Is the rehabilitation period in home-based care time-limited but extendable if needed/based on the goals for the patients with hip fracture and signs of cognitive impairment?

YES	
NO	

52. How are the rehabilitation sessions conducted at the home-based care for patients with hip fracture and signs of cognitive impairment?	
Group-based/classes	
Individual (one-on-one)	
Both options	

53. What modalities of exercise does a typical session consist of in home-based care for patients with hip fracture and signs of cognitive impairment? (Mark the category/categories that are relevant)	
Warm-up (e.g. on a stationary bike, walking with rollator))	
Cardiovascular exercise (BORG intensity level 14-15)	
Fitness (below BORG 14 intensity)	
Strength exercises	
Strengthening exercises (defined as more than +15 repetitions per exercise) (with weightcuffs, strength training machines, free weights, etc)	
Balance exercises (static or dynamic)	

Skill/functional tasks (flights, in/out of bed, up from floor)	
Exercises of relevant activities of daily living (ADL)(bathing, getting dressed, eat/drinking)	
Outdoor walking (with or without walking aid)	
Other	

54. Which tests do you use for patients with hip fracture and signs of cognitive impairment who receive their rehabilitation in home-based care? (Mark the relevant test(s))	
Mini-Mental State Examination (MMSE)	
Montreal Cognitive Assessment (MoCA)	
Brief Assessment of Impaired Cognition Questionaire (BASIC-Q)	
Clock Drawing Test	
Patient Specific Functional Scale (PSFS)	
De Morton Mobility Index (DEMMI)	
Cumulated Ambulation Score (CAS)	
New Mobility Score (NMS)	
Timed Up&Go (TUG)	
10-Meter Walking Test	
6-Minute Walk Test	
Verbal Rating Scale (VRS)	
Numeric Rang Scale (NRS)	
Visual Analog Scale (VAS)	
Borg Rating of Perceived Exertion	
Tandem test (balance)	
Berg´s Balance Scale	
Falls Efficacy Scale - International (FES-I) (16-64 point scale)	
Short Falls Efficacy Scale - International (sFES-I) (7-28 point scale)	
30-second Sit-To-Stand test	
Five Times Sit-To-Stand test	
Barthel Index	
Repetition Maximum (RM)	
0-5 muscle grading test	
Isometric muscle testing (e.g. with a dynamometer)	
Nothing	

Rehabilitation and testing in patients with hip fracture and signs of cognitive impairment at outpatient healthcare centers.

55. Who primarily conducts the rehabilitation of patients with hip fracture and signs of cognitive impairment who receive exercise at an outpatient healthcare center? (Mark the relevant category)	
Physiotherapist	
Occupational Therapist	
Both Physiotherapist and Occupational Therapist	
Physiotherapy Assistant	
Other	

56. How often do patients with hip fracture and signs of cognitive impairment exercise, who receive rehabilitation at an outpatient healthcare center? (Mark the relevant category)	
Fewer than 1 session/week	
1-2 sessions/week	
3-4 sessions/week	
Daily	
Several times daily	

57. What is the typical overall time frame (in minutes) for a single exercise session for patients with hip fracture and signs of cognitive impairment receiving rehabilitation at an outpatient healthcare center? (Mark the time interval that indicates the maximum duration of a session)	
0-15 min.	
16-30 min.	
31-45 min.	
46-60 min.	
61-75 min.	
+76 min.	

58. What is the typical overall time frame (in weeks) for the total period of rehabilitation for patients with hip fracture and signs of cognitive impairment receiving rehabilitation at an outpatient healthcare center? (Mark the week interval that typically indicates the total number of exercise weeks)	
0-2 weeks	
3-4 weeks	
5-6 weeks	
7-8 weeks	
9-10 weeks	
11-12 weeks	

13+ weeks	

59. Is the rehabilitation period at the outpatient healthcare center time-limited but extendable if needed/based on the goals for the patients with hip fracture and signs of cognitive impairment?	
YES	
NO	

60. How are the rehabilitation sessions conducted at the outpatient healthcare center for patients with hip fracture and signs of cognitive impairment?	
Group-based/classes	
Individual (one-on-one)	
Both options	

61. What modalities of exercise does a typical session consist of at an outpatient healthcare center for patients with hip fracture and signs of cognitive impairment? (Mark the category/categories that are relevant)	
Warm-up (e.g. on a stationary bike, walking with rollator))	
Cardiovascular exercise (BORG intensity level 14-15)	
Fitness (below BORG 14 intensity)	
Strength exercises	
Strengthening exercises (defined as more than +15 repetitions per exercise) (with weightcuffs, strength training machines, free weights, etc)	
Balance exercises (static or dynamic)	
Skill/functional tasks (flights, in/out of bed, up from floor)	
Exercises of relevant activities of daily living (ADL)(bathing, getting dressed, eat/drinking)	
Outdoor walking (with or without walking aid)	
Other	

62. Which tests do you use for patients with hip fracture and signs of cognitive impairment who receive their rehabilitation at an outpatient healthcare center? (Mark the relevant test(s))	
Mini-Mental State Examination (MMSE)	
Montreal Cognitive Assessment (MoCA)	
Brief Assessment of Impaired Cognition Questionaire (BASIC-Q)	
Clock Drawing Test	
Patient Specific Functional Scale (PSFS)	
De Morton Mobility Index (DEMMI)	

Cumulated Ambulation Score (CAS)	
New Mobility Score (NMS)	
Timed Up&Go (TUG)	
10-Meter Walking Test	
6-Minute Walk Test	
Verbal Rating Scale (VRS)	
Numeric Rang Scale (NRS)	
Visual Analog Scale (VAS)	
Borg Rating of Perceived Exertion	
Tandem test (balance)	
Berg´s Balance Scale	
Falls Efficacy Scale - International (FES-I) (16-64 point scale)	
Short Falls Efficacy Scale - International (sFES-I) (7-28 point scale)	
30-second Sit-To-Stand test	
Five Times Sit-To-Stand test	
Barthel Index	
Repetition Maximum (RM)	
0-5 muscle grading test	
Isometric muscle testing (e.g. with a dynamometer)	
Nothing	

Rehabilitation and testing of patients with hip fracture and with signs of cognitive impairment residing at nursing home facilities.

63. Who primarily conducts the rehabilitation of patients with hip fracture and signs of cognitive impairment who receive exercise in a nursing home facility? (Mark the relevant category)	
Physiotherapist	
Occupational Therapist	
Both Physiotherapist and Occupational Therapist	
Physiotherapy Assistant	
Other	

64. How often do patients with hip fracture and signs of cognitive impairment exercise, who receive rehabilitation in in a nursing home facility? (Mark the relevant category)	
Fewer than 1 session/week	
1-2 sessions/week	

3-4 sessions/week	
Daily	
Several times daily	

65. What is the typical overall time frame (in minutes) for a single exercise session for patients with hip fracture and signs of cognitive impairment receiving rehabilitation in a nursing home facility? (Mark the time interval that indicates the maximum duration of a session)	
0-15 min.	
16-30 min.	
31-45 min.	
46-60 min.	
61-75 min.	
+76 min.	

66. What is the typical overall time frame (in weeks) for the total period of rehabilitation for patients with hip fracture and signs of cognitive impairment receiving rehabilitation in a nursing home facility? (Mark the week interval that typically indicates the total number of exercise weeks)	
0-2 weeks	
3-4 weeks	
5-6 weeks	
7-8 weeks	
9-10 weeks	
11-12 weeks	
13+ weeks	

67. Is the rehabilitation period at the nursing home facility time-limited but extendable if needed/based on the goals for the patients with hip fracture and signs of cognitive impairment?	
YES	
NO	

68. How are the rehabilitation sessions conducted at the nursing home facility for patients with hip fracture and signs of cognitive impairment?	
Group-based/classes	
Individual (one-on-one)	
Both options	

69. What modalities of exercise does a typical session consist of at a nursing home facility for patients with hip fracture and signs of cognitive impairment? (Mark the category/categories that are relevant)	
Warm-up (e.g. on a stationary bike, walking with rollator))	
Cardiovascular exercise (BORG intensity level 14-15)	
Fitness (below BORG 14 intensity)	
Strength exercises	
Strengthening exercises (defined as more than +15 repetitions per exercise) (with weightcuffs, strength training machines, free weights, etc)	
Balance exercises (static or dynamic)	
Skill/functional tasks (flights, in/out of bed, up from floor)	
Exercises of relevant activities of daily living (ADL)(bathing, getting dressed, eat/drinking)	
Outdoor walking (with or without walking aid)	
Other	

70. Which tests do you use for patients with hip fracture and signs of cognitive impairment who receive their rehabilitation at a nursing home facility? (Mark the relevant test(s))	
Mini-Mental State Examination (MMSE)	
Montreal Cognitive Assessment (MoCA)	
Brief Assessment of Impaired Cognition Questionaire (BASIC-Q)	
Clock Drawing Test	
Patient Specific Functional Scale (PSFS)	
De Morton Mobility Index (DEMMI)	
Cumulated Ambulation Score (CAS)	
New Mobility Score (NMS)	
Timed Up&Go (TUG)	
10-Meter Walking Test	
6-Minute Walk Test	
Verbal Rating Scale (VRS)	
Numeric Rang Scale (NRS)	
Visual Analog Scale (VAS)	
Borg Rating of Perceived Exertion	
Tandem test (balance)	
Berg´s Balance Scale	
Falls Efficacy Scale - International (FES-I) (16-64 point scale)	
Short Falls Efficacy Scale - International (sFES-I) (7-28 point scale)	
30-second Sit-To-Stand test	

Five Times Sit-To-Stand test	
Barthel Index	
Repetition Maximum (RM)	
0-5 muscle grading test	
Isometric muscle testing (e.g. with a dynamometer)	
Nothing	

71. Do you have a formal description of the rehabilitation to patients with hip fractures and signs of cognitive impairment in your municipality?	
YES	
NO	
If YES, please send it to Jan Overgaard at jover@lolland.dk	
