# Supplementary material for: EXERCISE MODALITIES AND OUTCOME MEASURES USED IN OLDER ADULTS AFTER HIP FRACTURE WITH OR WITHOUT SIGNS OF COGNITIVE IMPAIRMENT: A NATIONAL CROSS-SECTIONAL E-SURVEY OF 90 OUT OF 98 MUNICIPALITIES IN DENMARK
Source: J Rehabil Med. 2026 Feb 11;58:44207. doi: 10.2340/jrm.v58.44207 (PMC12914642; doi:10.2340/jrm.v58.44207)
Supplement: Supplementary file 2 [file JRM-58-44207-s2.pdf]

Supplementary material has been published as submitted. It has not been copyedited, or typeset by Journal of Rehabilitation Medicine

Table SI. Twenty-five commonly reported outcome measures in patients with hip fracture (HF) and with signs of cognitive impairment (HF-SCI) in Danish municipalities.

**Outcomes**	**Abbreviations**
Patient Reported Outcomes – Cognitive assessment	
The Mini Mental State Examination	MMSE
The Montreal Cognitive Assessment	MoCA
Brief Assessment of Impaired Cognition Questionaire	BASIC-Q
Clock Drawing Test	Clock Drawing Test
Patient Reported Outcomes - Pain	
Verbal Rating Scale	VRS
Numeric Rating Scale	NRS
Visual Analogue Scale	VAS
Patient Reported Outcome - Activity	
Patient Specific Functional Scale	PSFS
New Mobility Score	NMS
Barthel Index	BI
Fall Efficacy Scale – International (long: 16-64 points)	FES-I(l)
Fall Efficacy Scale – International (short: 7-28 points)	FES-I(s)
Borg Rating of Perceived Exertion	BORG (RPE)
Performance-based scores/outcomes	
De Morton Mobility Index	DEMMI
Cumulated Ambulation Score	CAS
Timed Up&Go test	TUG
10-Meter Walking Test	10MWT
6-Minute Walk Test	6MWT
Short Physical Performance Test, balance element test	Tandem
Berg´s Balance Scale	BBS
30-second Sit-To-Stand test	30s STS
Five Times Sit-To-Stand test	5x STS
Repetition Maximum	RM
0-5 muscle grading test	0-5 test
Isometric muscle testing	Isometric

Table SII. Reported setting and distribution (in percentage) of rehabilitation offered to patients with hip fracture (HF) and with HF and signs of cognitive impairment (HF-SCI).

		HF				HF-SCI			
Municipality setting and number of respondents		24-hour care facilities, *n* = 89	Home-based care, *n* = 90	Outpatient healthcare facilities, *n* = 90	Nursing home facilities, *n* = 89	24-hour care facilities, *n* = 89	Home-based care, *n* = 90	Outpatient healthcare facilities, *n* = 90	Nursing home facilities, *n* = 89
Who is/are primarily responsible for the treatment/therapy?	Physiotherapist (PT)	57%	66%	94%	57%	36%	52%	79%	55%
	Occupational therapist (OT)	0%	3%	0%	4%	1%	2%	0%	5%
	PT & OT	42%	30%	6%	34%	61%	44%	21%	36%
	Other *	1%	1%	0%	5%	2%	2%	0%	4%
Distribution of session delivered/ offered	Less than 1/week	0%	3%	1%	7%	0%	3%	0%	6%
	1-2/week	33%	91%	98%	78%	33%	89%	98%	79%
	3-4/week	40%	6%	1%	8%	37%	6%	2%	9%
	Daily	25%	0%	0%	6%	26%	2%	0%	5%
	Multiple times daily	2%	0%	0%	2%	4%	0%	0%	1%
	0-15 min.	0%	0%	0%	1%	2%	2%	0%	2%

Distribution of the length provided per session	16-30 min.	25%	14%	2%	42%	34%	16%	4%	39%
	31-45 min.	58%	62%	26%	49%	54%	61%	32%	47%
	46-60 min.	17%	23%	69%	8%	10%	21%	61%	12%
	61-75 min.	0%	0%	3%	0%	0%	0%	3%	0%
Distribution of the time period of rehabilitation provided	0-2 weeks	8%	3%	0%	2%	6%	3%	1%	2%
	3-4 weeks	48%	10%	0%	4%	33%	2%	0%	3%
	5-6 weeks	17%	24%	6%	23%	22%	23%	7%	24%
	7-8 weeks	14%	29%	34%	28%	10%	34%	37%	27%
	9-10 weeks	4%	10%	12%	10%	14%	12%	11%	11%
	11-12 weeks	6%	18%	39%	26%	12%	20%	34%	26%
	+13 weeks	3%	6%	9%	7%	3%	6%	10%	7%
Distribution of how the intervention sessions is provided	Individual	62%	97%	7%	84%	69%	98%	22%	89%
	Group-based	0%	0%	30%	0%	0%	0%	19%	0%
	Individual & group-based	38%	3%	63%	16%	32%	2%	59%	11%
Notes: *Other = Nursing assistant.									

**Table SIII.** Reported distribution and type of test´s used (in percentage) during the rehabilitation in the municipalities								
	Patients with hip fracture				Patients with hip fracture and signs of cognitive impairment			
Municipality setting, *n*	24-hour care facilities, *n* = 89	Home-based care, *n* = 90	Outpatient healthcare centers, *n* = 90	Nursing home facilities, *n* = 89	24-hour care facilities, *n* = 89	Home-based care, *n* = 90	Outpatien healthcare centers, *n* = 90	Nursing home facilities, *n* = 89
Patient reported outcome (PROM) cognitive assessments								
MMSE	7%	2%	1%	1%	16%	8%	4%	2%
MoCA	19%	10%	3%	6%	28%	16%	13%	11%
BASIC-Q	0%	1%	1%	0%	0%	1%	1%	0%
Clock drawing test	2%	0%	2%	0%	5%	2%	2%	1%
PROM Pain								
VRS	8%	9%	11%	8%	10%	8%	6%	5%
NRS	40%	41%	44%	34%	26%	28%	26%	26%
VAS	21%	22%	20%	14%	15%	11%	14%	9%
PROM Activity								
PSFS	16%	26%	30%	18%	15%	17%	19%	12%
NMS	9%	14%	16%	10%	9%	11%	11%	8%

Barthel Index	10%	8%	1%	5%	8%	1%	1%	2%
FES-I (16-64 point)	1%	1%	8%	0%	0%	1%	2%	1%
FES-I (7-28 point)	1%	2%	2%	1%	1%	1%	1%	0%
BORG	6%	6%	11%	1%	1%	1%	1%	1%
Performance-based scores								
DEMMI	33%	20%	8%	14%	26%	13%	9%	12%
CAS	15%	12%	13%	13%	15%	13%	11%	8%
TUG	56%	38%	71%	26%	38%	26%	57%	27%
10MWT	21%	7%	28%	10%	18%	4%	21%	11%
6MWT	34%	19%	68%	14%	21%	9%	50%	12%
Tandem	44%	49%	52%	25%	28%	27%	40%	18%
BBS	11%	9%	17%	5%	5%	8%	9%	7%
30s STS	74%	73%	90%	63%	63%	71%	79%	56%
5x STS	6%	7%	6%	6%	5%	4%	8%	3%
RM	9%	8%	16%	5%	5%	3%	4%	5%
0-5 test	34%	41%	53%	30%	34%	29%	37%	29%
Isometric	3%	1%	7%	2%	2%	1%	4%	1%
No testing	8%	7%	2%	18%	16%	20%	10%	31%

Abbreviations: MMSE = The Mini Mental State Examination, MoCA = The Montreal Cognitive Assessment, BASIC-Q = Brief Assessment of Impaired Cognition Questionaire, VRS = Verbal Rating Scale, NRS = Numeric Rating Scale, VAS = Visual Analogue Scale, PSFS = Patient Specific Functional Scale, NMS = The New Mobility Score, FES-I (16-64 point) = The Falls Efficacy Scale – International (long: 16-64 points), FES-I (7-28 points) = Fall Efficacy Scale – International (short: 7-28 points), BORG = Borg Rating of Perceived Exertion, DEMMI = De Morton Mobility Index, CAS = The Cumulated Ambulation Score, TUG = The Timed Up&Go test, 10MWT = 10-Meter Walking Test, 6MWT = 6-Minute Walk Test, Tandem = Short Physical Performance Test, balance element test, BBS = Berg´s Balance Scale, 30s STS = 30 second Sit-To-Stand test, 5x STS = Five Times Sit-To-Stand test, RM = Repetition Maximum, 0-5 Test = 0-5 muscle grading test, Isometric = Isometric muscle testing.

Table SIV. Reported number of sessions offered (per week) to patients with hip fracture (HF) and with HF and signs of cognitive impairment (HF-SCI).

	Municipality setting, *n*	1-2 times/week, *n* (%)	3-4 times/week, *n* (%)	All weekdays, *n* (%)
HF	Outpatient healthcare centers, *n* = 90	90 (100)	0 (0)	0 (0)
	Home-based care, *n* = 90	85 (94)	5 (6)	0 (0)
	24–hour care facilities, *n* = 89	29 (33)	36 (40)	24 (27)
	Nursing home facilities, *n* = 89	75 (84)	7(8)	7 (8)
HF-SCI	Outpatient healthcare centers, *n* = 89	87 (98)	2 (2)	0 (0)
	Home-based care, *n* = 90	83 (92)	5 (6)	2 (2)
	24-hour care facilities, *n* = 89	29 (33)	33 (37)	27 (30)
	Nursing home facilities, *n* = 89	75 (84)	8 (9)	6 (7)

Note: Data are expressed as numbers (*n*) in percentage (%) of municipalities responding.

Table SV. Reported duration of sessions (in minutes) offered to patients with hip fracture (HF) and with HF and signs of cognitive impairment (HF-SCI).

	Municipality setting, *n*	0-30 min., *n* (%)	31-45 min., *n* (%)	46-60 min., *n* (%)
HF	Outpatient healthcare centers, *n* = 90	2 (2)	23 (26)	65 (72)
	Home-based care, *n* = 90	13 (15)	56 (62)	21 (23)
	24-hour care facilities, *n* = 89	22 (25)	52 (58)	15 (17)
	Nursing home facilities, *n* = 89	13 (15)	56 (62)	21 (23)
HF-SCI	Outpatient healthcare centers, *n* = 89	4 (5)	28 (31)	57 (64)
	Home-based care, *n* = 90	16 (18)	55 (61)	19 (21)
	24-hour care facilities, *n* = 89	33 (37)	47 (53)	9 (10)
	Nursing home facilities, *n* = 89	37 (42)	40 (45)	12 (13)

Note: Data are expressed as numbers (*n*) in percentage (%) of municipalities responding.

Table SVI. Reported duration of rehabilitation offered to patients with hip fracture (HF) and with HF and signs of cognitive impairment (HF-SCI).

	Municipality setting, *n*	0-4 weeks, *n* (%)	5-8 weeks, *n* (%)	9-12 weeks, *n* (%)	+13 weeks, *n* (%)
HF	Outpatient healthcare centers, *n* = 90	0 (0)	36 (40)	46 (51.1)	8 (8.9)
	Home-based care, *n* = 90	12 (13.3)	47 (52.2)	26 (28.9)	5 (5.6)
	24-hour care facilities, *n* = 89	50 (56.2)	27 (30.3)	9 (10.1)	3 (3.4)
	Nursing home facilities, *n* = 89	6 (6.7)	45 (50.6)	32 (36)	6 (6.7)
HF-SCI	Outpatient healthcare facilities, *n* = 89	1 (1.1)	39 (43.8)	40 (44.9)	9 (10.1)
	Home-based care, *n* = 90	5 (5.6)	51 (56.7)	29 (32.2)	5 (5.6)
	24-hour care facilities, *n* = 89	34 (38.2)	29 (32.6)	23 (25.8)	3 (3.4)
	Nursing home facilities, *n* = 89	5 (5.6)	45 (50.6)	33 (37.1)	6 (6.7)

Note: Data are expressed as numbers (*n*) in percentage (%) of municipalities responding.
